# Supplementary material for: Hopes, joys and fears: Meaning and perceptions of viral load testing and low-level viraemia among people on antiretroviral therapy in Uganda: A qualitative study
Source: PLOS Glob Public Health. 2023 May 10;3(5):e0001797. doi: 10.1371/journal.pgph.0001797 (PMC10171654; doi:10.1371/journal.pgph.0001797)
Supplement: S1 Table — (DOCX) [file pgph.0001797.s001.docx]

**Hopes, Joys and Fears: Meaning and perceptions of viral load testing and low-level viraemia among people on antiretroviral therapy in Uganda: A qualitative study**

**Codebook**

| **Code** | **Definition/Description** | **Example** |
| --- | --- | --- |
| A non-detectable viral load is preferred | Having a viral load not detected is better | The good thing would be 0 detection of the HIV virus |
| Acceptance and positive living | Accepting your HIV situation and starting to live positively | Please accept that you are positive and no matter how you got it |
| Alcohol can affect viral load | Effects of alcohol on viral load | Drink much alcohol and eat less because that is what causes the viral load to get to a non-suppressed state |
| Appointment dates not given to PLHIV at times | PLHIV not told when to return for viral load testing at times | They don’t inform us. Last year in the month of May, I had my blood sample taken up to now, I have not had my blood taken |
| Avail transport | Give transport to PLHIV | They can provide transport |
| Availability of viral load requirements | Presence of what to use to do viral load testing | Basically, the first one is the availability of the required utilities. |
| Avoid alcohol | Not taking alcohol | They should avoid drinking alcohol and take their drugs on time |
| Avoiding stress | Stopping things that worry you | Don’t listen to those that gossip with statements that worry you |
| Being represented at the clinic by other people | A PLHIV sending another person to pick for them the drugs | And for children and youth most of them send their parents to pick from them their drugs |
| Cannot get low-level viraemia | Do not think they can get LLV | I don’t think I will get low-level viraemia. |
| Communal support | Any support or help received from the community | The community can encourage that person |
| Community/home based services | HIV services offered to PLHIV in their community or at their homes | That person is tested at their home |
| Conflicting priorities | Any other activities which competes with the PLHIV’s time to go for viral load testing | I had gone for a funeral |
| Continuous reminders and help from health workers to do VL | Health workers to remind and support the PLHIV to do viral load | These healthcare providers need to remind them |
| Delayed HIV Diagnosis | Failure to detect that a person has HIV early enough | It took approximately four years when sickness was disturbing me |
| Delayed viral load results | Results not coming back in time | We need those things  (results) to come back faster |
| Desire to be healthy | Do viral load in order to be healthy | I want to become healthy again which is the reason I am dedicated to it |
| Diagnosed with other infections/diseases | Having other infections/diseases | The healthcare providers simply found malaria |
| Difficult getting to the health facility | Living so far from health facility, and finding it hard to reach the facility | They live far from here and getting to the health facility is a big challenge |
| Disappointed in viral load services | Feeling disappointment with VL services | They usually say that they are tired of testing for viral load without  getting their test results |
| Disorganization at the Clinic | Having the clinic mixed up with so many things and patients not cared after | You can be tossed up and down by medical workers |
| Do not know what viral load means | Not understanding what viral load testing means | Others don’t really know what viral load testing means |
| Don’t know what viral load is | Not knowing what viral load is | I don’t understand it so well |
| Don't know what LLV means | Cannot tell what LLV is, and confesses that they do not know what it means | Low-level viraemia…I don’t know and I don’t want to lie that I would explain it. |
| Drugs and viral load prolong life | Taking drugs and doing viral load testing increases one’s lifetime | Get your drugs and do your viral load tests. This is the most important thing, because it can help extend their days of living |
| Drugs not working well | The drugs not helping the PLHIV to suppress HIV | A particular drug may not work for you completely |
| Drugs reduce the viral load | The drugs help to decrease viral load | Drug that will fight the virus and make sure that it weakens it thereby reducing the viral load copies |
| Early return of viral load results | Viral load results coming back early | So if there is a way for which the clients get their test results so quick just like for HIV testing |
| Encouragement for taking drugs | Motivation for taking drugs, as a result of viral load | It’s also easy for one to take drugs with an understanding that the viral load copies are reducing |
| Family support | Any support received from the family | The family should also give a helping hand to that person who is living with HIV on ART |
| Family/communal challenges | Various problems at family and communal level | This can begin from home and homes have collection of problems and you can easily forget your |
| Fear and stigma for HIV | Communal fear and discrimination associated with HIV | A lot of people in the communities that gossip about people living with HIV |
| Fear coming for treatment and viral load testing | Being scared of coming to the health facility | Most of them fear coming here to receive their drugs and viral load testing |
| Feel peaceful | A feeling of being at ease, and having a sense of being at peace | I have found peace unlike before |
| Few health workers | Small number of health workers at the clinic | Exactly, the health workers are not enough |
| Follow medical advice | Doing what the health workers tell you | They should follow advice from the healthcare providers |
| Getting alternative transport means | Using any possible transport means to go to the health facility | I usually hire a bicycle from someone which I use for coming here |
| Give patients appointment dates | Writing dates to return for viral load for the patients | The health workers normally write the date in your book; this acts like a reminder |
| Good diet | Eating a well-balanced diet | It is important for one to care about their health first by taking their meals on time, eat greens and make sure that they take some balanced diet |
| Happy with good viral load results | A feeling of happiness when the viral load results are good/suppressed | For those whose results are good, they will be happy |
| Have myths | Any myths | But some of them have myths |
| Help detect new illnesses | Viral load can help to know if the person has another disease | Doctor can tell that there is another illness starting in your body and it can easily be dealt with |
| High viral load is caused by drug resistance or poor adherence | Drug resistance and not taking drugs well can cause high viral load | When your viral load is high sometimes you may have drug resistance or you are not following the health workers guidelines. |
| High viral load is not good | High viral load having negative effects | The viral load copies are more, they will die quickly |
| I have the ability | Having the ability and capability to do viral load testing | I have the right and the ability to keep showing up for the viral load test |
| Information on viral load testing | Any information or document on viral load testing | The healthcare providers have given me a written document indicating that I should always come for viral load testing |
| Know my viral load status | A PLHIV knowing their viral load results and status | I know my viral load suppression status |
| Know the process of viral load testing | Being able to tell the process of viral load sample collection and testing | They take off your blood if you are due, and send it to the laboratory for testing |
| Know whether drugs is working | Viral Load helps to tell whether the drugs are working or not | This will enable the doctor to know whether the drugs are working or not |
| Know whether patient is taking drugs well | Viral load helps health workers to know whether the patient is taking the drugs well | You should be tested to see how you have been taking your drugs |
| Lack of appetite | Not having appetite to eat food | I could not even eat |
| Lack of enough counselling | PLHIV not getting enough counselling | Such people have not received enough counselling |
| Lack of privacy at ART/HIV clinic | There is privacy at the HIV clinic, and there are other people | There are those for the art department who sit right here, those with dental issues are also right there, and you realize that there is no privacy |
| Lack of transport | Having no transport | I also have a challenge with  transport |
| Level of virus in blood | Viral load means the amount/level of HIV in blood | It helps you to know whether the viral copies have gone high or they have lowered |
| Long acting drugs | Drugs which are not swallowed daily | I wish they can get for us drug which you can take once in a month… |
| Long distances | Travelling long distances | That particular challenge of long distance |
| Long waiting time for services | PLHIV having to wait for long hours at facilities | They sit until they get up and escape back home without receiving healthcare |
| Looking for transport money | Means of trying to find money for transport to the health facility | I have to look for money early enough |
| Loss of hope | Words showing loss of hope | Some people have given up on their lives |
| Low confidence in HIV testing | Lack of trust in the HIV testing services | Those machines can show positive results yet I am negative |
| Low viral copies cannot increase again in blood | Low-level viraemia is not risk for an increased viral load | Low-level viraemia cannot multiply and go higher again |
| Low viral load improves life and health | The sense that a low viral load improves life and health | The viral load copies have gone down and I have strength |
| Low-level viraemia improves life | Having low-level viraemia is good, and it improves life | You will definitely stay strong and allow you add your days of living just like those that are not living with HIV because when the viral load goes high, you will grow weak as well |
| Low-level viraemia caused by missing drugs | Missing drugs causes low-level viraemia | I have had LLV, like I told you I used to over miss my drugs when I was  at school |
| Low-level viraemia has no effects | Low-level viraemia is not dangerous | I feel very fine |
| Low-level viraemia is a suppressed viral load | Misinterpretation of low-level viraemia as a suppressed viral load | I think the virus level in my body is low; so having a suppressed  virus in my blood is my future ambition |
| Low-level viraemia is not good and worrying | Any concerns or worries about low-level viraemia | It worries because the reason why I am saying that is that those seem to be the resistant copies and it most likely going to multiply depending on the regiment you are taking |
| Low-level viraemia means a decreased and good viral load | Misinterpretation of low-level viraemia as a low and good viral load | When you do your viral load testing routinely and also take your drugs in time, your viral load copies will decrease. That is what low-level viraemia is all about |
| Many PLHIV do not know that viral load is a right | People not being aware that viral load is their right | I don’t think they are aware that viral load is their right |
| Misinterpretation of a suppressed VL as a non-suppressed VL | Mixing up suppressed and non-suppressed viral loads | If the drug is working, but mine is working very well and my result came back when it was non- suppressed |
| Misinterpretation of LLV as a non-suppressed VL | Thinking that low-level viraemia is a non-suppressed VL | You have high viral load but have not heard about it personally |
| Misinterpretation of low-level viraemia as the HIV infection | Thinking that low-level viraemia is the same as being infected with HIV | I have heard about it but what I have not heard is that someone has cured completely |
| Misinterpretation of viral load as CD4 | Thinking that viral load is the same as CD4 | The viral copies will lower completely. And you also will begin to grow weak |
| Missing appointments | Not coming back to the facility on the appointment date | Someone missing appointment and failing to pick their drugs |
| Missing viral load results | Not finding your viral load results at the health facility | You may come here and find that your viral load results have been misplaced appointment |
| Monitor health | Viral load helps to track one’s health status | I am also able to monitor my health easily |
| More healthy | Having no complaints of regular sickness | I no longer feel sickly |
| Multiple partners is risky and worsen viral load | Having many sexual partners is dangerous, and can worsen the viral load | Don’t go and engage in sexual activities because you will create an environment to increase |
| Necessary to test viral load | It is very useful to do a viral load test | It is important that one should take drugs as well as having his/her viral load tested |
| Need extra government support | Any other support other than medical care needed from the government | But let the government help us in any way possible |
| Need more staff at the ART clinic | The need to have more health workers at the ART clinic | I think the number should be increased because you know |
| No communal support for appointments | No follow up in the community to remind PLHIV of their appointments | We do not have such a person, we do not have anyone |
| No problems with viral load | Not facing any challenges with viral load testing | I have not faced any challenges so far |
| Non-disclosure and confidentiality | Not telling other people about one’s HIV status and lack of privacy | Those that do not want people to know that they are living with HIV |
| Not taking drugs well | Failing to take the drugs well as directed by the health worker | If you just disappear and fail to take your drugs in a right way |
| Now live life normally | Being able to live and do anything, just like any other person | I can also go to the garden to dig normally |
| Offer Intensive adherence counselling | Offer intensive adherence counselling to PLHIV with a high viral load | They put you to adherence counselling |
| Old and aged PLHIV, who cannot move | Old age affecting the ability to go to the health facility | That particular challenge of long distance is mixed with old age as well |
| Overworking can affect viral load results | Overworking can lead to a high viral load | Your viral load is high probably because you are over working yourself |
| Patient follow-up | Following up the PLHIV to come back for viral load services | You simply remind him/her to come and pick your drugs or do a viral load test |
| Patients should demand for viral load | PLHIV asking health workers to do their viral load tests | When you go to the doctor’s room you ask like the other time you took blood from me, so what were the results? |
| Peer support | PLHIV supporting each other in any ways | Sometimes we assist  each other especially with food stuff when need arise to some of our friends who are so weak |
| Poor diet can affect viral load | Not eating well can cause a high viral load | Your viral load is high probably because you are over working yourself and you don’t eat well |
| Poverty is a big challenge | Not having money to use | Sometimes I have to look for money and then hire someone to come plough for me. That is the only problem otherwise when it comes to physical wellbeing, I am very fine |
| Praying to reduce viral load | Spiritual intervention to lower viral load | Also, prayer can help lower the viral load |
| Private and secret clinics for HIV care and viral load | Secret clinics to provide HIV services and viral load services | They want to be given their own place which is very secret and safe |
| Reduce waiting time for services | Ensure that patients do not wait for long | People can easily be worked on quickly before they get exhausted |
| Regularly review my appointment date | Routinely look at my appointment date, not to miss my return to the facility | I will always keep checking on the book until the date of appointment |
| Routine sickness | Getting regular sicknesses and diseases | I often felt a lot of body pain, weakness, I felt my body aching frequently |
| Sad with bad viral load results | Feeling sad and unhappy when the viral load results are bad or non-suppressed | For those with non-suppressed viral load results, you will find them with closed eyes, very sad and angry |
| Scared of dying | Fear to die | I may cut my days short and I may get to die early before my time |
| Scared of viral load | A feeling of fear to do viral load testing | Some people are always scared of going for a viral load test |
| Self-denial | Not accepting that the person is HIV positive | It seems you are the one who has contacted the HIV virus and not me |
| Sensitization, counselling and health education | The need to counsel and educate PLHIV on viral load testing | Those people need a lot of counselling |
| Some PLHIV cannot read to know when to return for appointment | Inability to read, thereby missing the appointments | We are managing but others can’t because they can’t read |
| Some PLHIV do not care about viral load testing | PLHIV are negligent about doing viral load testing | Some people are just not bothered about viral load testing |
| Stress affects viral load | Worrying can cause a high viral load | When you start worrying too much, you will take the drugs but it will seem like you have not taken it or you will forget taking them. |
| Take caution, not transmit HIV | Being careful not transmit HIV to other people | I have managed to safeguard my family from contacting the HIV virus. I don’t allow sharing sharp instruments with them |
| Taking drugs well | Swallowing the drugs well as directed | Secondly, keep time for taking drugs |
| Tough health workers | Cruel or unkind health workers | They take my blood and then you  guys shout at me |
| Track my viral load suppression status | Following up with one’s viral load status | I desire so much to see the change and keep track of my viral load suppression status |
| Urgently seek medical care | Get healthcare urgently | They feel unwell or any complications in their health, they should rush to the healthcare facility |
| Use of peers to remind PLHIV of their appointments | Peer mentors helping to follow up with the PLHIV | Recruit others who will always remind them when they are supposed to return for the next appointments |
| Viral load guides the next treatment steps | Making treatment decisions after getting a viral load result | If the viral copies are high, the healthcare providers will advise you on what to do |
| Viral load inspires a change in bad behaviour | A person changing behaviour due to viral load | while others also leave over thinking because when they find oooooh, the virus is increasing in your blood you have to struggle very hard to see that you leave behind all bad  habits like alcohol, a lot of thoughts among others , so that you adhere on drug , you start living  in proper way |
| Viral load is a right | The PLHIV knowing that he/she has a right to do viral load testing | I have the capability and the right to do my viral load because this is my life |
| Viral load is non-suppressed | When the viral load is high, and not good | I was tested and found that my viral load was non-suppressed |
| Viral load is suppressed | A viral load that is below 1000 copies, or PLHIV indicating that the viral load is suppressed | I am very strong and my viral load is suppressed |
| Viral load means testing blood | When blood samples are taken off, to test for viral load | It is when the some substances or blood samples are taken from you and taken for testing |
| Viral load tells a doctor when to change the drug | The decision to change the drugs may be done after viral load testing | If the healthcare providers get to know that your viral load is high, they can see how to change the drugs you are taking |
| Weight loss | Loosing body weight | If you always weighed twenty seven for example, this time round you may weigh twenty four |
| Willingly come for viral load testing | Coming to test for viral load willingly | I left my home willingly to come and have myself tested for my own health issues |
| Would prefer doing viral load more frequently | Desire to do viral load more often, than usual | I feel if it was possible to even perform these tests every single week |
