# Supplementary material for: Hopes, joys and fears: Meaning and perceptions of viral load testing and low-level viraemia among people on antiretroviral therapy in Uganda: A qualitative study
Source: PLOS Glob Public Health. 2023 May 10;3(5):e0001797. doi: 10.1371/journal.pgph.0001797 (PMC10171654; doi:10.1371/journal.pgph.0001797)
Supplement: Attachment — Submitted filename: Responses to Reviewers.pdf [file pgph.0001797.s002.pdf]

Dr. Nanyeenya Nicholus,
Makerere University School of Public Health,
New Mulago Hill Road, Mulago Kampala.
Tel: +256 774 734803 / +256 704 734803
Email: nanyeenya@gmail.com

March 17, 2023

The Academic Editor and Reviewers,
PLOS Global Public Health.

Dear Sirs/Madams,

REF: Responses to the review comments for the Manuscript titled, 'Hopes, Joys and Fears: Meaning and perceptions of viral load testing and low-level viraemia among people on antiretroviral therapy in Uganda: A qualitative study.'

First and foremost, we greatly and humbly thank you all for taking off time to carefully read our manuscript and giving us very insightful review comments. We greatly believe that addressing these comments has been very key in improving our manuscript. Thank you very much once again.

We hereby humbly submit the responses to the different review comments raised, as shown below;

1. ACADEMIC EDITOR

Thank you for submitting your manuscript to PLOS Global Public Health. After careful consideration, we feel that it has merit but does not fully meet PLOS Global Public Health's publication criteria as it currently stands. Therefore, we invite you to submit a revised version of the manuscript that addresses the points raised during the review process.

a) Please review your reference list to ensure that it is complete and correct. If you have cited papers that have been retracted, please include the rationale for doing so in the manuscript text, or remove these references and replace them with relevant current references. Any changes to the reference list should be mentioned in the rebuttal letter that accompanies your revised manuscript. If you need to cite a retracted article, indicate the article's retracted status in the References list and also include a citation and full reference for the retraction notice.

   - Thank you very much for this very useful comment. We have read through the entire manuscript again carefully looking at every reference following your guidance. We have realized that some of the references had not been updated automatically by the referencing software, and hence we have updated them. A number of references including; 2, 11, 12, 13, 30, 31, 33, 34, 36 and 37 have been

updated to reflect the exact articles that they reference, as shown in the manuscript with track changes. We are sorry for this oversight, and we greatly thank you very much once again for noticing this oversight in the manuscript. Thank you very much.

b) Your manuscript is missing the following sections: Introduction. Please ensure these are present, and in the correct order, and that any references to subheadings in your main text are correct. An outline of the required sections can be consulted in our submission guidelines here: https://journals.plos.org/globalpublichealth/s/submission-guidelines#loc-parts-of-a-submission

- Thank you very much for this key observation. We have read the submission guidelines again, and we have corrected this, as shown in Line 74 on page 4.

c) We have noticed that you have uploaded Supporting Information files, but you have not included a list of legends. Please add a full list of legends for your Supporting Information files after the references list.

- Thank you for this observation. This has been addressed, as shown in Lines 716 to 717 on Page 27. Thank you very much.

d) In the online submission form, you indicated that "The codebook for the study has been availed. Any further datasets used and/or analysed during the current study are available from the corresponding author on reasonable request". All PLOS journals now require all data underlying the findings described in their manuscript to be freely available to other researchers, either 1. In a public repository, 2. Within the manuscript itself, or 3. Uploaded as supplementary information.
This policy applies to all data except where public deposition would breach compliance with the protocol approved by your research ethics board. If your data cannot be made publicly available for ethical or legal reasons (e.g., public availability would compromise patient privacy), please explain your reasons by return email and your exemption request will be escalated to the editor for approval. Your exemption request will be handled independently and will not hold up the peer review process, but will need to be resolved should your manuscript be accepted for publication. One of the Editorial team will then be in touch if there are any issues.

- Thank you very much for this. We have addressed this in the online submission.

2. REVIEWER #1

General Comment

HIV management is one aspect of the field of medicine that has faced a lot of challenges. Initial challenges revolved around acceptance of HIV status, stigma, lack of support systems, and availability of ARVs especially in low and middle-income countries. Emerging challenges are issues to do with adherence to medication and the management of HIV in general. The author's findings on HIV patient perception towards VL testing is a plus in the management of HIV. The ability of PWH to interpret and appreciate the role of LLV in HIV management is a good sign of the acceptability of VL testing by the patients in their management. The conclusion of the study identify areas that needs improvement in the utilization of VL testing in the monitoring and management of HIV patients.

2.1 Major Points

a) To deal with sampling bias during participant selection, the authors should clarify if they include defaulters in their sample (pg 6).

- Thank you very much for this comment. It also actually raises ethical concerns and we have addressed this in Lines 132 to 133 on page 6. Thank you for raising this comment. Furthermore, we are also humbly informing you that we used stratified purposive sampling (as shown in Line 127 on page 6) to enable us get the participants who were fit to answer the study research questions in details. Thank you very much.

b) One aspect of low-level viremia is misinterpretation and misuse of the LLV results by the patients themselves or by other support systems around the patients. How did the authors capture this challenge (pg 17-18)

- Thanks for this comment, and it is actually an insightful challenge. However we discussed this challenge further in the discussion section, from Lines 502 to 508 on Page 21. Thank you very much.

c) Was culture a barrier to VL testing? (pg 14)

- Thank you very much for this question. Of course, culture has always been a barrier, and several interventions like sensitization have been used to overcome it. In this study, we used an inductive approach, which involved hearing more from what the participants said, compared to what we knew (deductive approach). In the interviews, the participants talked about different myths, though some of them were not really related to culture as such. However we greatly agree with you that culture is still a barrier in HIV care, which needs to be investigated thoroughly well.

2.2 Minor Points

d) The background has a focus on Uganda only, it will be better if the authors can bring forth what is happening in other jurisdictions.

- Thank you very much. It would be good to look at what happens elsewhere however we desired to focus on Uganda in this study, to give a better context for our study. Furthermore, similar studies have not been conducted elsewhere in Sub-Sahara Africa, which has similar settings like Uganda. Thanks very much.

e) Mention accessibility of VL testing in Uganda and African countries.

- Thank you very much for this insightful comment. This has been addressed as shown in Lines 86 to 88 on Page 4. Thanks very much.

f) It would have been better if the authors included the healthcare givers in their study population. Generally, healthcare provision is a collaborative effort between the patients and the clinicians.

- Thank you for noting this. The perceptions of health workers about LLV and VL testing would be great to include here. However the manuscript would be large, and the study team agreed to do this later on, and include it in another totally separate manuscript. Thank you very much.

3. REVIEWER #2

Nanyeenya et. al present a qualitative study based on the health belief model in which they assess the perceptions of HIV viral load testing and low-level viremia in people living with HIV in Uganda. This study provides unique insights into patient perceptions and understanding of viral load testing and results interpretation. Such studies are a crucial piece needed for developing more effective interventions to improve the overall care for people with HIV in a manner that centers their needs and enhances their understanding of these interventions. The manuscript is very well written and clearly presented. There are some comments the authors may wish to consider to improve on their work.

a) In the discussion I would have liked to see the authors discuss how their results could be used to design interventions which can improve on how patients view low level viremia and even propose plausible interventions that could be effective based on the data they have generated.

- Thanks very much for raising this very critical comment. This has been addressed as shown in Lines 497 to 500, 529 to 530, and 532 to 534 in the discussion section. Once again, thank you for highlighting this comment.

b) I applaud the efforts made by the authors to methodically conduct detailed interviews adapted for language and cultural setting which is so crucial to obtain information that accurately represents the views of the respondents however the sample size (n=32 detailed interviews) of the study is quite small which makes one to wonder whether such a small sample size is sufficient to fully capture the full spectrum of perceptions among people living with HIV. Could the authors comment on this choice of sample size and acknowledge this limitation in the manuscript?

- Thank you very much for this comment. In order to make this study feasible, we estimated that a minimum of 32 in-depth interviews (IDIs) with PLHIV on ART would be conducted until information saturation was reached. This minimum sample size was estimated based on Saunders et al., (Saunders et al., 2018) and Guest et al., (Guest et al., 2006) where we anticipated that by the time the 32 IDIs would be finished, there would be no new data obtained from the participants. Furthermore, stratified purposive sampling (Palinkas et al., 2015), was used to select the participants for the study, from both rural and urban settings, considering both male and female sex, level of education, and age to create diversity and variability in the study.
  Below are the references;
  1. *Saunders B, Sim J, Kingstone T, Baker S, Waterfield J, Bartlam B, et al. Saturation in qualitative research: exploring its conceptualization and operationalization. Qual Quant. 2018;52(4):1893–907.*
  2. *Guest G, Bunce A, Johnson L. How Many Interviews Are Enough?: An Experiment with Data Saturation and Variability. Field Methods. 2006 Feb 1;18(1):59–82.*
  3. *Palinkas LA, Horwitz SM, Green CA, Wisdom JP, Duan N, Hoagwood K. Purposeful sampling for qualitative data collection and analysis in mixed method implementation research. Adm Policy Ment Health. 2015 Sep;42(5):533–44.*

c) The authors present their results very well. A summary figure or table of the key themes emerging from the detailed interviews as detailed in the sub-headings in the results section will break the monotony of lengthy text and provide a nice summary of the findings to accompany the detailed reporting of the results.

- Thank you very much for this very insightful and critical comment. We have developed summary figure as per your guidance, as shown by the caption on Line 188 on page 8. The figure has also been saved and uploaded as Fig 1, as required by the Journal guidelines.

d) Line 541 – minor typo correct behaviours currently spelled as bahaviors

- Thank you very much for this. We have addressed it as shown in Line 546.

Once again, thank you very much for the comments.

Yours faithfully,

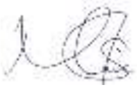

Dr. Nanyeenya Nicholus.
